# P05 Cellulitis uncovered: a retrospective dive into cellulitis management

**DOI:** 10.1093/jacamr/dlaf118.012

**Published:** 2025-07-14

**Authors:** Anagha Vasant, Samyojana, Ayesha, Pooja

**Affiliations:** St Peter’s and Ashford Hospital, Surrey, UK; St Peter’s and Ashford Hospital, Surrey, UK; St Peter’s and Ashford Hospital, Surrey, UK; St Peter’s and Ashford Hospital, Surrey, UK

## Abstract

**Background:**

Cellulitis is a painful and potentially serious infection of the skin and underlying tissue affecting approximately 1 in 40 people per year- resulting in 100 000 admissions in hospital in UK each year. The Dundee classification system and NICE guidelines provide a framework for managing cellulitis, emphasizing appropriate classification, narrow spectrum antibiotics for 5-7 days. This audit evaluates current cellulitis management practices and identifies deviations from these standards.

**Objectives:**

(i) To assess treatment adherence to Dundee classification system and trust guidelines; (ii) to identify causes of prolonged antibiotic use (>7 days); and (iii) to propose a revised pathway tailored to improve patient outcome.

**Methods:**

A retrospective review of 200 patients treated with cellulitis in 2023 was conducted. The clinical management was analysed against the Dundee classification and trust guidelines with the focus on treatment duration, risk stratification and reoccurrence management.

**Results:**

*Adherence to classification:* 56% of cases were not concordant with the Dundee classification. *Overtreatment in class 1:* 39% of class 1 were overtreated, with 10.9% experiencing significant antibiotic side effects. *Undertreatment in class 2 and 3:* 56% of class 2 and 3 were undertreated, leading to 71% recurrence rate.

**Recommendations:**

(i) Implement a revised pathway combing trust guidelines with a modified Dundee classification system to improve grading accuracy and guide treatment decisions. (ii) Include diabetes and lymphedema as risk factors in the modified Dundee classification. (iii) Identify high risk patients and initiate the prophylactic antibiotic therapy to prevent recurrent admissions. (iv) Conduct a follow-up audit to evaluate the effectiveness of the proposed changes.

**Conclusions:**

This audit highlights significant deviation from the recommended cellulitis management guidelines, resulting in both overtreatment and undertreatment. The proposed interventions aim to optimize care, minimize recurrence and reduce adverse antibiotics effects. A follow-up audit will assess the impact these improvements.

